# Supplementary material for: MetaRibo-Seq measures translation in microbiomes
Source: Nat Commun. 2020 Jun 29;11:3268. doi: 10.1038/s41467-020-17081-z (PMC7324362; doi:10.1038/s41467-020-17081-z)
Supplement: Supplementary file 10 — Supplementary Data 7 [file 41467_2020_17081_MOESM10_ESM.zip › File2/Confidence_High_Taxonomy/58442_out.krona.html]

Javascript must be enabled to view this page.

members
magnitude
magnitudeUnassigned
count
unassigned
taxon
rank

58442\_out

17

superkingdom
2
17

phylum
17
976

17
200643
class

171549
17
order

family
1
815

genus
816
1

1
46506

SRS018888\_contig\_number\_9099
species

family
171552
16

genus

SRS023526\_contig\_number\_contig-100\_3049.223896
1
838
16

species
165179
10

SRS012969\_contig\_number\_38148SRS012989\_contig\_number\_7375SRS015782\_contig\_number\_contig-100\_3281.217749SRS015794\_contig\_number\_6688SRS045713\_contig\_number\_21660SRS046502\_contig\_number\_9483SRS053398\_contig\_number\_11406SRS077194\_contig\_number\_11459SRS077849\_contig\_number\_29909SRS146764\_contig\_number\_contig-100\_369.196735

species
59823
3

SRS014415\_contig\_number\_contig-100\_528.27381SRS144506\_contig\_number\_contig-100\_237.312959SRS150029\_contig\_number\_13217

2
159272

SRS103987\_contig\_number\_2567SRS144603\_contig\_number\_17104
species
